# Cabozantinib (XL184) and R428 (BGB324) Inhibit the Growth of Esophageal Squamous Cell Carcinoma (ESCC)

**DOI:** 10.3389/fonc.2019.01138

**Published:** 2019-11-06

**Authors:** Pei-Wen Yang, Yu-Cheng Liu, Ya-Han Chang, Ching-Ching Lin, Pei-Ming Huang, Kuo-Tai Hua, Jang-Ming Lee, Min-Shu Hsieh

**Affiliations:** ^1^Department of Surgery, National Taiwan University Hospital and National Taiwan University College of Medicine, Taipei, Taiwan; ^2^Graduate Institute of Toxicology, College of Medicine, National Taiwan University, Taipei, Taiwan; ^3^Graduate Institute of Pathology, College of Medicine, National Taiwan University, Taipei, Taiwan; ^4^Department of Pathology, National Taiwan University Hospital and National Taiwan University College of Medicine, Taipei, Taiwan

**Keywords:** esophageal cancer (EC), esophageal squamous cell carcinoma (ESCC), cabozanitinb, R428, targeted therapy

## Abstract

Esophageal squamous cell carcinoma (ESCC) is a deadly disease for which no effective targeted therapeutic agent has been approved. Both AXL and c-MET have been reported to be independent prognostic factors for ESCC. Thus, inhibitors of AXL/c-MET might have great potential as targeted therapy for ESCC. In the current study, we evaluated the therapeutic potential of the AXL/c-MET selective inhibitors, R428 and cabozantinib, in cell and mouse xenograft models. We demonstrated that both R428 and cabozantinib significantly inhibited the growth of CE81T and KYSE-70 ESCC cells and showed by wound-healing assay that they both inhibited ESCC cell migration. In the animal model, ESCC xenograft models were established by injecting KYSE-70 cells with Matrigel into the upper back region of NOD-SCID male mice followed by treatment with vehicle control, R428 (50 mg/kg/day), cisplatin (1.0 mg/kg), or cabozantinib (30 mg/kg/day) for the indicated number of days. R428 alone significantly inhibited ESCC tumor growth compared to the vehicle; however, no synergistic effect with cisplatin was observed. Notably, the dramatic efficacy of cabozantinib alone was observed in the mouse xenograft model. Collectively, our study demonstrated that both cabozantinib and R428 inhibit ESCC growth in cell and xenograft models. The results reveal the great potential of using cabozantinib for targeted therapy of ESCC.

## Introduction

Esophageal squamous cell carcinoma (ESCC) accounts for over 90% of primary esophageal cancer, a deadly disease with poor prognosis. ESCC is prevalent mostly in non-Caucasian male populations, predominantly in certain Asian, African, and South American areas (1). Patients usually remain undiagnosed until reaching a locally advanced stage as no obvious symptoms present at early stages of the disease. The standard treatment of locally advanced ESCC is (pre-operative) concurrent chemoradiotherapy (CCRT) followed by surgery (2, 3). Good response to CCRT has been found to correlate with better prognosis (4); however, the pathologically complete remission rate is usually <30% (5, 6). Regrettably, there is no approved targeted therapeutic strategy available to ESCC patients who opt not to receive CCRT or respond poorly to it (7). Although esophageal cancer survival rates have improved in recent years, the prognosis of ESCC is still poor. The overall 5-year survival rate is usually <20% even with multiple treatment modalities (1, 8, 9).

Receptor tyrosine kinases (RTKs) are a subclass of growth factor receptors on the cell surface that are endowed with tyrosine kinase activity controlled by growth factor ligands (10). RTKs regulate multiple functions in cells, including cell proliferation, survival, and differentiation, and numerous tyrosine kinase inhibitors (TKIs) have been approved by the FDA for targeted therapy (11, 12). HER2/Neu RTK is a drug target for gastroesophageal (GE) junction adenocarcinoma (13). To what extent the efficacy of the HER2 targeted inhibitors might carry over to ESCC is uncertain. We previously demonstrated that AXL (also known as Ark or Ufo), a member of the TAM (TYRO3, AXL, MER) family of RTKs, is a strong adverse prognostic factor for ESCC (14). A multi-kinase inhibitor, foretinib (GSK1363089), known to target AXL, c-Met, and VEGFR-2, exhibited a significant inhibitory effect, both alone and synergistically with HER2 inhibitors (14). The c-MET RTK is also an independent prognostic factor for ESCC (15, 16). Overexpression of c-MET, but not HER2 or epidermal growth factor receptor (EGFR), has been correlated with poor prognosis in ESCC (16). A c-MET inhibitor significantly inhibited the invasive activity in ESCC cells under the stimulation of its ligand HGF (15). All of this evidence suggests that inhibitors targeting AXL and c-MET have great potential as targeted therapeutics for ESCC.

R428 (bemcentinib, BGB324), a selective small-molecule inhibitor of AXL, is currently being evaluated in phase II trials for the treatment of non-small-cell lung cancer (NSCLC) (17) and acute myelocytic leukemia (AML). It has been found to induce apoptosis in cancer cells (18) and to block tumor spread in models of metastatic breast cancer (19). The therapeutic potential of R428 has also been demonstrated in highly invasive esophageal adenocarcinoma cells and in ESCC cells (20, 21).

Cabozantinib (XL184, Cabometyx^TM^, BMS-907351) is an oral multi-targeted small-molecule TKI, which targets VEGFR, MET and AXL. It has been approved in the USA to treat advanced renal cell carcinoma (RCC) and locally advanced or metastatic medullary thyroid cancer (MTC) (22, 23). The anti-tumor activity of cabozantinib has also been demonstrated in preclinical studies for breast cancer (24, 25), prostate cancer (25, 26), hepatocellular carcinoma (HCC) (27, 28), lung cancer (29), and bladder cancer (30). Its efficacy has also been demonstrated in phase II clinical trials for NSCLC (17, 31) and in phase III trials for advanced HCC (32). BMS-777607 is also a selective oral inhibitor of the MET kinase superfamily (33). Its efficacy has been demonstrated in a MET-dependent human gastric carcinoma (33).

In the current study, we evaluated these selective small-molecule inhibitors targeting AXL and c-MET, including R428, cabozantinib, and BMS-777607, in cell and mouse xenograft models of ESCC.

## Materials and Methods

### Cell Cultures

CE81T/VGH (CE81T) and KYSE-70 are human ESCC cell lines derived from a Taiwanese and Japanese population, respectively (34, 35). CE81T and KYSE-70 cells were cultured in DMEM/F12 and RPMI complete medium, respectively, supplemented with 10% FBS, and were maintained in a 37°C incubator containing 5% CO2.

### Inhibitors and Survival Assay

The AXL- and MET-related small-molecule inhibitors, R428 (BerGenBio ASA), BMS-777607 (Bristol-Myers Squibb), and cabozantinib (Exelixis), were purchased from and synthesized by ApexBio Taiwan (Hsinchu, Taiwan), Selleckchem (Houston, USA), and AdooQ BioScience (Irvine, USA), respectively. The efficacy of each inhibitor in inhibiting ESCC cell growth was analyzed by MTT survival assay as described previously (36). The sigmoidal dose–response curve and half maximal inhibitory concentration (IC50) were generated and analyzed by Graph-Pad Prism software (Graph-Pad software, Inc.). The combination index (CI) was calculated by CompuSyn software (CompuSyn, Inc.).

### Protein Extraction and Western Blotting

The protein expression profiles of cells in response to drug treatment were analyzed by western blotting as described previously (37). Briefly, total protein was extracted from the cells or tumor tissues by using RIPA buffer and separated by SDS-PAGE followed by western blotting. The primary antibodies for western blotting were anti-AXL polyclonal antibody (pAb) (#8661, Cell Signaling Technology [CST]), anti-phospho-AXL (Tyr702, D12B2, CST) monoclonal antibody (mAb) (#5724, CST), anti-MET mAb (#8198, CST), anti-phospho-MET mAb (#3126, CST), anti-MMP1 mAb (#MAB901, R&D systems), anti-ERK 1 (K-23) pAb (Santa Cruz), anti-phospho-p44/42 Erk1/2 (Thr202/Tyr204) mAb (#4370, CST), anti-Akt pAb (#9272, CST), anti-phospho-Akt pAb (Ser473) (#9271, CST), anti-actin mAb (clone C4, Millipore), and anti-α-tubulin mAb (DM1A, Abcam). Anti-E-cadherin mAb (#14472, CST), anti-vimentin mAb (#5741, CST), and anti-N-cadherin mAb (#13116, CST) were used for analysis of epithelial-to-mesenchymal transition (EMT). The signal intensities were analyzed by ImageQuant 5.1 (Molecular Dynamics, Inc.).

### ESCC Xenograft Model

The *in vivo* animal study has been approved by the Ethics Committee for Laboratory Animal Research of National Taiwan University. KYSE-70 cells (1.5 × 10^6^) with Matrigel (Corning) were subcutaneously injected into the upper back region of 6- to 8-week-old NOD-SCID male mice. In the R428 experiment, mice were randomly divided into four groups. The mice were treated with vehicle (0.5% hydroxypropylmethylcellulose + 0.1% Tween 80 in H_2_O); R428 (50 mg/kg/day, oral gavage); cisplatin (1.0 mg/kg), a dose previously shown to be non-therapeutic alone (38), every other day, by intraperitoneal injection; or both R428 and cisplatin. In the cabozantinib experiment, mice were randomly divided into two groups. The mice were treated with vehicle (65% H_2_O, 30% polyethylene glycol, and 5% Tween 80) or cabozantinib (30 mg/kg/day, oral gavage). All treatments started when the tumor volume reached around 100 mm^3^. Tumor size was measured twice a week using calipers. The tumor volume was calculated by the formula: 0.5 × (major axis) × (minor axis)^2^. The residual tumor tissues were snap-frozen for protein extraction.

### Wound Healing Assay

The procedures of the wound-healing assay (*in vitro* scratch assay) are mostly based on previous studies (38–40). CE81T cells were cultured in DMEM/F12 medium containing 2% FBS in six-well plates. At about 90% confluence, the cells were pre-treated with mitomycin C (Sigma) in serum-free medium, and then the monolayer was scraped to create a straight scratch with a p200 pipet tip. The debris and unbound cells were removed, and the remaining cells were then cultured in medium containing 2% FBS and either vehicle or indicated amounts of R428 or cabozantinib for 18, 24, 42, or 48 h. Images of the wounds at the indicated time points were visualized and captured by a light microscope (Nikon ECLIPSE TS100), and the wound healing rates were analyzed by ImageJ software.

## Results

### The Efficacy of R428, BMS-777607, and Cabozantinib in ESCC Cells

**Figure 1:**
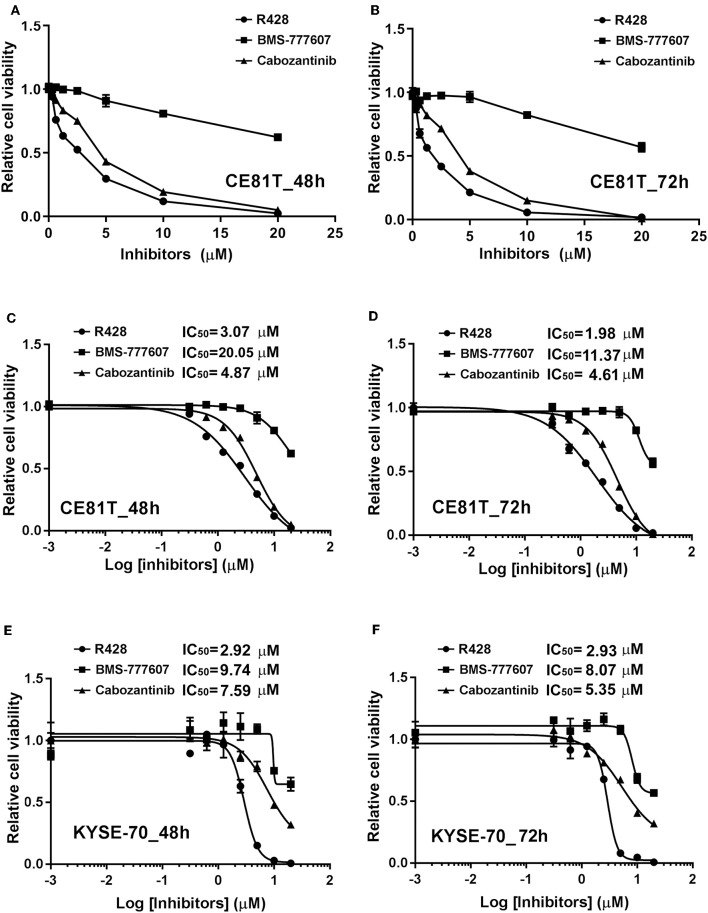
The efficacy of AXL and c-Met inhibitors in esophageal squamous cell carcinoma (ESCC) cells. Dose–response curves for cytotoxicity are displayed for CE81T cells in response to the inhibitors, including R428 (•), BMS-777607 (■), and cabozantinib (▴), at doses of 0.001, 0.3125, 0.625, 1.25, 2.5, 5, 10, and 20 μM after 48 h **(A)** and 72 h **(B)** of treatment. Sigmoidal dose-inhibition curves were constructed for CE81T **(A–D)** and KYSE-70 ESCC cells **(E,F)** in response to indicated amounts of indicated inhibitors at 48 h **(C,E)** and 72 h **(D,F)** post-treatment. The half maximal inhibitory concentration (IC50) values are exhibited as indicated.

In the current study, we first evaluated the efficacy of three potential AXL and c-MET small-molecule inhibitors in ESCC cells. Figure 1 exhibits the dose-dependent cytotoxicities of R428, BMS-777607, or cabozantinib treatment for 48 h (Figure 1A) or 72 h (Figure 1B) in CE81T ESCC cells. The cytotoxicity of R428 and cabozantinib was more evident compared to BMS-777607. The sigmoidal inhibitory dose–response curves in both CE81T and KYSE-70 cells were constructed. The IC50 of each inhibitor was determined. In CE81T cells, the IC50 values of R428 and cabozantinib were 1.98 and 4.61 μM, respectively, when treated for 72 h (Figure 1D). The IC50 of BMS-777607 could not be determined. Similar trends were observed in CE81T cells treated for 48 h (Figure 1C) and in KYSE-70 cells (Figures 1E,F).

**Figure 2:**
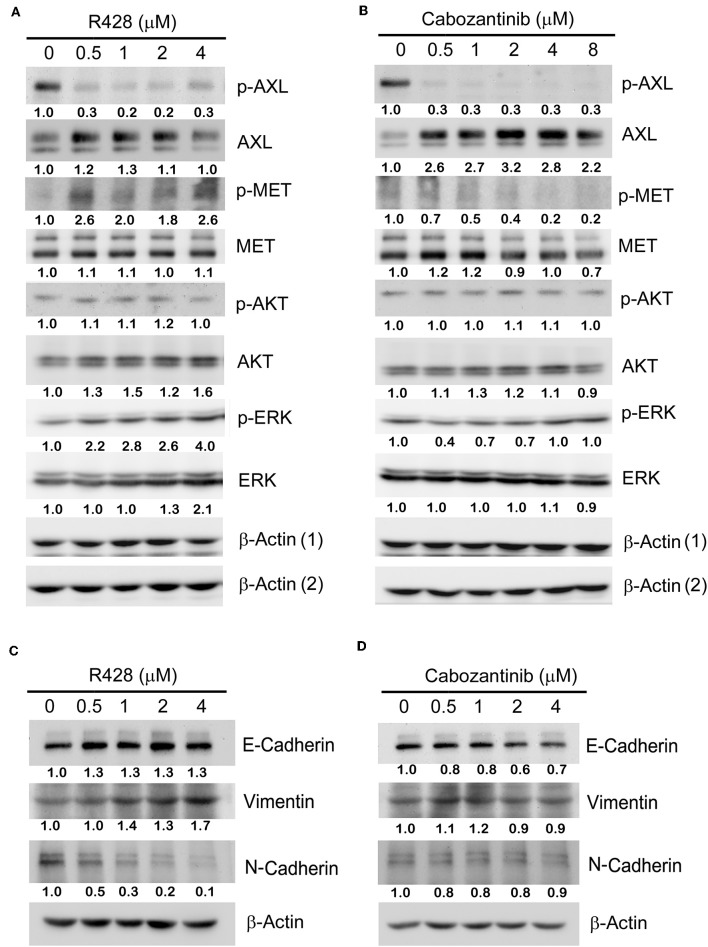
The protein expression profiles in CE81T cells treated with increasing amounts of R428 **(A)** or cabozantinib **(B)**. Expression of phospho-AXL (p-AXL), total AXL, p-AKT, total AKT, p-ERK, and total ERK were analyzed by western blotting by using specific antibodies. β-actin (1) served as a loading control for the groups analyzing p-AXL, p-AKT, and ERK. β-actin (2) served as a loading control for the groups analyzing AXL, AKT, and p-ERK. **(C,D)** The epithelial-to-mesenchymal transition (EMT) biomarker expression profiles in R428-treated **(C)** and cabozantinib-treated cells **(D)**. E-cadherin, vimentin, and N-cadherin were analyzed with specific antibodies. The relative intensities (normalized by loading control) relative to control group (0 μM) are indicated below the protein signals.

The protein expression profiles of CE81T cells treated with R428 (Figure 2A) and cabozantinib (Figure 2B) were analyzed by western blotting. Phospho-AXL was significantly decreased in response to both R428 and cabozantinib treatment even though total AXL was likely induced by both inhibitors. Phospho-MET seemed to be decreased with increased amounts of cabozantinib, whereas it increased with increased amounts of R428. Phospho-Akt was not significantly altered in response to both R428 and cabozantinib treatment. Both ERK and phospho-ERK (pERK) levels exhibited a significant increase after treatment with R428 (Figure 2A).

AXL has been frequently linked to EMT in some cancers, including esophageal cancer (41, 42). Reduced expression of the EMT marker Snail has been observed in R428-treated primary tumors in an animal model (19). Cabozantinib has also been demonstrated to partially suppress EMT induced by TGF-β in breast tumor cells (43). Thus, we then analyzed the expression profile of the EMT markers in response to drug treatment in esophageal cancer cells. The basal level of E-cadherin was abundant in CE81T cells and was only slightly increased in R428-treated cells. N-cadherin was decreased dose-dependently, whereas vimentin was slightly increased in response to R428 treatment (Figure 2C). Vimentin and N-cadherin levels did not significantly change after cabozantinib treatment. E-cadherin was slightly decreased in cells treated with higher amounts of cabozantinib (Figure 2D).

### The Combined Treatment of Cisplatin With R428 or Cabozantinib in ESCC Cells

**Figure 3:**
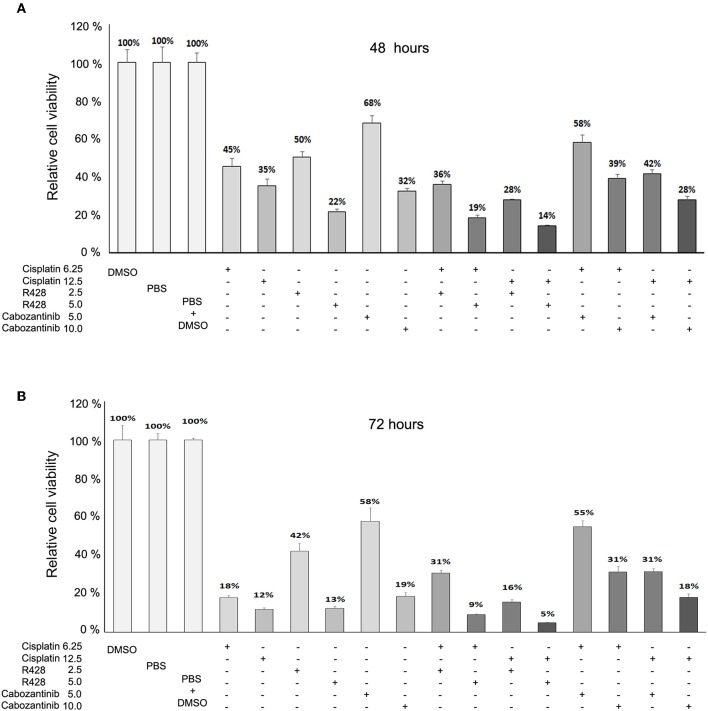
Effects of R428 (2.5 or 5 μM) and cabozantinib (5 or 10 μM) alone or combined with cisplatin (6.25 or 12.5 μM) on CE81T ESCC cells at 48 h **(A)** and 72 h **(B)** post-treatment. The relative viability is the cell survival rate relative to solvent control (PBS or DMSO).

We also analyzed whether cisplatin enhanced the efficacy of R428 or cabozantinib. We found that cisplatin might exert a synergistic effect with a higher dose of R428 (5 μM) but not with cabozantinib (CI = 0.53 and 0.041 for cells treated with R428 plus 6.25 or 12.5 μM of cisplatin, respectively, Figure 3A). However, the synergism of R428 and cisplatin was not significant when cells were treated for 72 h (CI>1, Figure 3B).

### The Effect of Cabozantinib and R428 on Migration Activity of ESCC Cells

**Figure 4:**
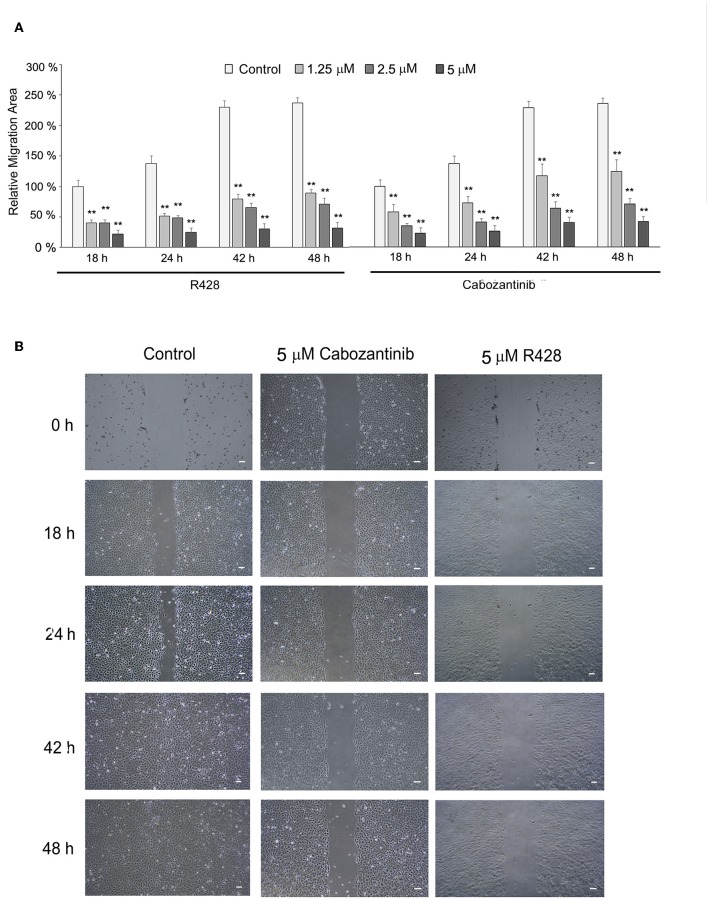
The effect of cabozantinib and R428 on ESCC cell migration analyzed by wound-healing assay. **(A)** The relative migration area of the cells treated with indicated amounts of cabozantinib or R428 compared to the area of control cells at 18 h post-treatment. The differences in migrating area between control and drug-treated groups were analyzed by independent *t*-test. **P < 0.001. **(B)** The images of wound length at 0, 18, 24, 42, and 48 h (h) post-treatment with DMSO (control), 5 μM cabozantinib, or R428. Scale bar represents 100 μm.

R428 and cabozantinib have been found to reduce migration activity of cancer cells (39, 44). We further investigated their effect on ESCC cell migration by wound-healing assay (Figure 4). CE81T cells exhibited strong migration activity without stimulation. Both cabozantinib and R428 markedly suppressed the migration activity in CE81T cells significantly compared to control (Figure 4).

### Evaluation of the Efficacy of R428 and Cabozantinib in ESCC Xenograft Model in Mice

**Figure 5:**
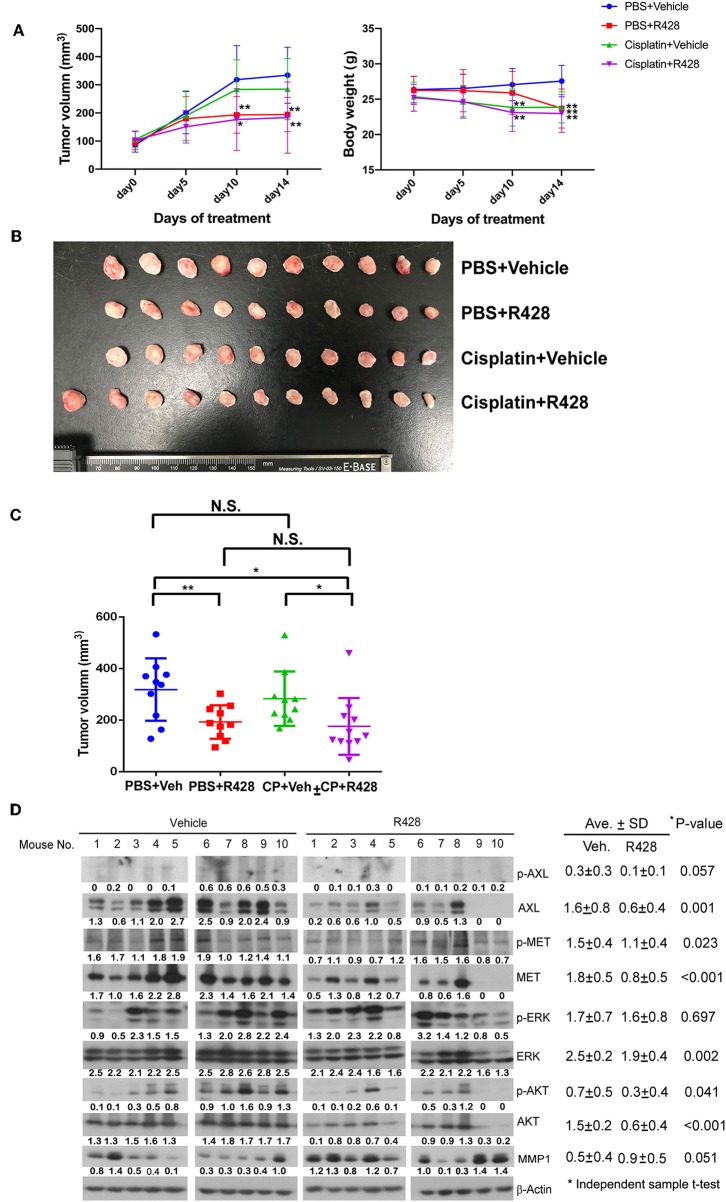
Assessment of R428 efficacy in an ESCC xenograft model. The ESCC tumor-bearing mice were vehicle-controlled (•, *N* = 10) or treated with R428 (■, *N* = 10, 50 mg/kg/day), cisplatin (▴, *N* = 10, 1.0 mg/kg), or cisplatin plus R428 (▾, *N* = 11). **(A)** Tumor volume (left) and body weight (right) of mice evaluated for 14 days in control and drug-treated groups. **(B)** Mouse xenografts at 14 days post-treatment. **(C)** The tumor volume distribution of mouse xenografts among control and drug-treated groups at 14 days post-treatment. **(D)** The protein expression profiles of mouse xenografts in vehicle or R428-treated group analyzed by western blotting with specific antibodies. β-actin served as a loading control. The intensities relative to loading control are indicated below the protein signals. *P < 0.05; **P < 0.01.

The efficacy of R428 was also evaluated in a mouse xenograft model (Figure 5). The tumor volume was analyzed in the vehicle control group and the groups treated for 14 days with R428, cisplatin, or R428 combined with cisplatin. The body weight of mice was significantly decreased in those treated with R428 alone at day 14 (Figure 5A) and was also significantly reduced in the cisplatin-treated group at days 10 and 14 post-treatment (*P* < 0.01, Figure 5A). In comparison with the vehicle control group, the average tumor growth rates were significantly decreased in the groups treated with R428 alone (*P* < 0.01 at day 10 and day 14, Figure 5A) and cisplatin combined with R428 (*P* < 0.05 at day 10 and *P* < 0.01 at day 14, Figure 5A). Figure 5B exhibits the ESCC xenografts at 14 days' treatment in each group. R428 significantly reduced the tumor volume compared to the vehicle control (*P* < 0.01, Figure 5C). However, cisplatin did not increase the efficacy of R428 compared to treatment with R428 alone (Figure 5C). We also analyzed the protein expression profile of each xenograft tissue sample. The expressions of total AXL and MET and their phospho- forms in the R428-treated group were generally less than in the vehicle group (*P* = 0.001 and 0.057 for AXL; *P* = 0.023 and <0.001 for MET). The average AKT and phospho-AKT levels were also less in R428-treated mice than in the controls (*P* < 0.001 and *P* = 0.041).

**Figure 6:**
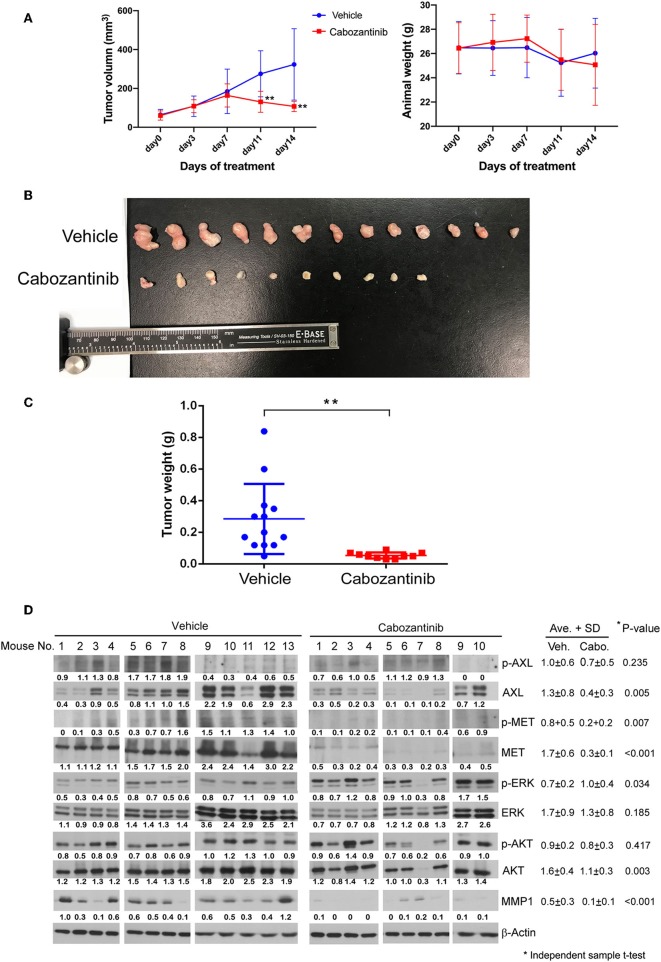
Evaluation of cabozantinib efficacy in an ESCC xenograft model. **(A)** Plots of 14-day growth curves of the mean tumor volume (left) and body weight of mice (right) in the vehicle control group (•, *N* = 13) and cabozantinib-treated group (■, *N* = 10, 30 mg/kg/day). **(B)** Mouse xenografts at 14 days post-treatment. **(C)** The weight distribution of the ESCC xenografts in the control group and cabozantinib-treated group at 14 days post-treatment. **(D)** The protein expression profiles of the mouse xenografts in the vehicle group and cabozantinib-treated group. β-actin served as a loading control. The intensities relative to loading control are indicated below the protein signals. *P < 0.05; **, *P* < 0.01.

The efficacy of cabozantinib was also demonstrated in the mouse study (Figure 6). Cabozantinib significantly inhibited the tumor growth of the ESCC xenografts (*P* < 0.01, Figure 6A). Body weight did not significantly decrease in the cabozantinib-treated mice compared to the vehicle group (Figure 6A). Both tumor size and weight were markedly decreased in the cabozantinib-treated group in comparison to the control (vehicle) group (*P* < 0.01, Figures 6B,C). Similar to the expression profiles in the R428-treated cells, AXL, phospho-MET, and MET were reduced in the cabozantinib-treated group compared to in the vehicle group (*P* = 0.005, 0.007, and <0.001 respectively). pERK expression was more pronounced in the drug-treated xenografts (*P* = 0.034, Figure 6D), a result that has previously been observed in neuroblastoma (45). Notably, the expression of matrix metalloproteinase-1 (MMP-1), a crucial factor in cell migration, was markedly decreased in the cabozantinib-treated mice compared to in the vehicle group (P <0.001, Figure 6D). However, decreased MMP-1 was not observed in the R428-treated xenografts (Figure 5D).

## Discussion

ESCC is a deadly disease for which no targeted agents have been approved. Erlotinib and gefitinib, the small-molecule inhibitors targeting EGFR, have been evaluated in esophageal cancer in several clinical trials. Response to erlotinib has been observed in 2 ESCC patients in a phase II trial with 13 cases (46). Gefitinib was used as a second-line treatment in unselected esophageal cancer patients in a phase III trial but did not improve overall survival (47). Although AXL is not considered a major oncogenic driver, its overexpression has been found to be correlated with a wide array of cancer-related events, including resistance to certain targeted agents and chemotherapy, tumor angiogenesis and metastasis, suppression of anti-tumor immune response, and EMT (17).

A growing number of AXL/c-MET multiple-target inhibitors have been developed. The efficacy of these inhibitors has been demonstrated in numerous preclinical and clinical studies (17). We previously demonstrated the prognostic role of AXL in ESCC and the potential of an AXL inhibitor in targeted therapy (14). In this continued study, we have demonstrated the efficacy of cabozantinib in inhibiting ESCC cell viability and migration activity (Figures 1, 4). No synergistic effect was found after adding cisplatin (Figure 3). The dramatic efficacy of cabozantinib alone was also demonstrated in an ESCC xenograft model (Figure 6). Cabozantinib has been reported to inhibit MMP-1 expression by blocking the HGF-MET signaling pathway in bladder cancer cells (30). Here we found that MMP-1 was significantly decreased in cabozantinib-treated xenografts, which could possibly explain the reduced migration activity of cabozantinib-treated ESCC cells. Cabozantinib has become a successful novel therapy for advanced MTC and RCC (22, 23). The FDA has also recently approved cabozantinib for treatment of advanced HCC (May 2018) based on a successful phase III study (32). Our study demonstrates the great potential of cabozantinib as a targeted agent for ESCC.

It has been reported that about 87% of ESCC tissue expresses c-MET (16). Overexpression of c-MET in ESCC has been found ranging from about 43 to 70% (15, 48). However, MET DNA amplification is rarely found in cancers (49, 50). We reported previously that almost 80% of ESCC tissue had positive staining for AXL (14). We also analyzed by FISH (fluorescence *in situ* hybridization) the AXL gene amplification in 18 ESCC tissue samples with strong positive AXL staining. Only one of them (1/18) had AXL gene amplification (polysomy; average AXL signals per cell: 9; AXL/CEN19 ratio: 1). Therefore, AXL gene amplification is probably not the major reason for AXL protein overexpression in ESCC tissues. Unlike other major targeted agents such as EGFR, HER2, and ALK inhibitors, there are still no reliable biomarkers to predict the treatment response to cabozantinib.

Our study demonstrated the efficacy of R428, but not of BMS-777607 (Figure 1), in inhibiting ESCC cell growth. Although BMS-777607 has been found to inhibit cell growth in a xenograft model of gastric carcinoma and to suppress the metastatic phenotype of HGF-induced prostate cancer (33, 51), we did not observe a significant effect of BMS-777607 on the viability of either CE81T or KYSE-70 ESCC cells (Figure 1). In addition to viability, R428 also suppressed the migration activity of ESCC cells (Figure 4). R428 alone also significantly decreased ESCC tumor growth compared to vehicle in the mouse model (Figure 5), however, not as remarkably as cabozantinib did. R428 has been reported to synergize with cisplatin to enhance the cell death of mesothelioma and to suppress liver micrometastasis of breast cancer (19, 52). We observed a synergistic effect of R428 and cisplatin on ESCC cells at 48 h post-treatment (Figure 3A). However, we were unable to demonstrate the synergistic activity at an extended incubation of 72 h (Figure 3B) or in the *in vivo* mouse model (Figure 5). In our mouse study, we analyzed the synergistic effect by choosing a dose of cisplatin (1.0 mg/kg) that did not show significant efficacy or an obvious effect on body weight but had shown a synergistic effect with other inhibitors according to the results of a previous study (38). In our experiment, such a low dose of cisplatin still significantly decreased the body weight of mice (10 and 14 days, Figure 5A); however, the enhancement of R428 efficacy was not observed. We thus speculate that cisplatin does not significantly enhance the efficacy of R428 in ESCC. There are several ongoing phase II clinical trials combining R428 and other targeted immunotherapeutic agents to treat malignant diseases, including NSCLC, breast cancer, melanoma, and acute myeloid leukemia. The search for an appropriate drug that can be combined with R428 to enhance its efficacy in treating ESCC needs further exploration in a future preclinical study.

We previously observed that foretinib induces AXL expression in ESCC cells (14). In the current study, we found that treatment with R428 or cabozantinib also increased AXL expression in the cell model (Figure 2). We believe that this might be due to feedback stimulation of the AXL pathway since the phosphorylation of AXL was dramatically decreased in the drug-treated cells. However, expression of AXL and MET were both obviously eliminated in the drug-treated xenograft model (Figures 5, 6). Since cancers usually have a high proportion of proliferative cells and are associated with a hypoxic environment, inhibition of tumor growth by AXL/MET inhibitors might decrease their oxygen demand. Therefore, the microenvironment of xenograft tumors may become less hypoxic, resulting in down-regulation of AXL and MET.

AXL has been known to play an essential role in inducing EMT (53). However, in R428-treated cells, atypical EMT was observed. The EMT marker vimentin was up-regulated, while N-cadherin exhibited decreased expression with an increased amount of R428 (Figure 2C). EMT has frequently been reported to be correlated with TKI resistance (54, 55). Since the protein was extracted from the viable cells under drug treatment, drug-resistant cells might possibly have been isolated. And therefore, some of the EMT biomarkers were observed. Cadherin switching plays an important role in tumorigenesis and cancer prognosis (56). Decreased N-cadherin may reduce invasion and growth of tumor cells during drug treatment. Treatment with cabozantinib did not significantly alter the EMT phenotype of the ESCC cells (Figure 2D). Thus, the functional mechanism of cabozantinib in ESCC cells may be not mediated by EMT inhibition.

In conclusion, our study is the first to demonstrate the efficacy of cabozantinib and R428 in both ESCC cell and xenograft models. This preclinical study reveals the great potential of cabozantinib as a targeted therapy for ESCC. The clinical efficacy and related biomarkers for the most beneficial treatment of ESCC with cabozantinib need to be further explored in future clinical studies.

## Data Availability Statement

All datasets for this study are included in the manuscript/supplementary material.

## Ethics Statement

This study was carried out in accordance with the recommendations of the Ethics Committee for Laboratory Animal Research of National Taiwan University. The protocol was approved by the Ethics Committee for Laboratory Animal Research of National Taiwan University.

## Author Contributions

P-WY, M-SH, and J-ML provided the concept and design of the study and analyzed the data. P-WY performed the literature search and wrote the manuscript. M-SH and J-ML revised the manuscript. Y-CL, Y-HC, and C-CL performed experiments and part of the literature search. P-MH and K-TH provided research resources.

### Conflict of Interest

The authors declare that the research was conducted in the absence of any commercial or financial relationships that could be construed as a potential conflict of interest.
